# Experimental Study on Proportion Optimization of Rock-like Materials Based on Genetic Algorithm Inversion

**DOI:** 10.3390/ma17194940

**Published:** 2024-10-09

**Authors:** Hui Su, Shaoxing Liu, Baowen Hu, Bowen Nan, Xin Zhang, Xiaoqing Han, Xiao Zhang

**Affiliations:** 1 College of Water Conservancy and Hydropower, Hebei University of Engineering, Handan 056038, China; suh-26@163.com (H.S.); lsx_13292783080@163.com (S.L.); 13313181477@163.com (B.N.); 15732292386@163.com (X.Z.); 2 Hebei Key Laboratory of Intelligent Water Conservancy, Handan 056038, China; 3 Provincial and Ministerial Collaborative Innovation Center for Intelligent Regulation and Comprehensive Management of Water Resources, Handan 056038, China; 4 Jizhong Energy Fengfeng Group Co., Ltd., Handan 056038, China; yipianyuye@163.com; 5 College of Water Resources and Environment, China University of Geosciences, Beijing 100083, China; 16603179466@163.com

**Keywords:** rock-like materials, orthogonal test, multiple linear regression, genetic algorithm, ratio optimization

## Abstract

It is very important to clarify the optimization method of the rock-like material ratio for accurately characterizing mechanical properties similar to the original rock. In order to explore the optimal ratio of rock-like materials in gneissic granite, the water–paste ratio, iron powder content and coarse sand content were selected as the influencing factors of the ratio. An orthogonal test design and sensitivity analysis of variance were used to obtain the significant influencing factors of the ratio factors on seven macroscopic mechanical parameters, including compressive strength *σ_c_*, tensile strength *σ_t_*, shear strength *τ_f_*, elastic modulus *E*, Poisson’s ratio *ν*, internal friction angle *φ* and cohesion *c.* A multivariate linear regression equation was constructed to obtain the quantitative relationship between the significant ratio factors and the macroscopic mechanical parameters. Finally, a rock-like material ratio optimization program based on genetic algorithm inversion was written. The results show that the water–paste ratio had extremely significant effects on *σ_c_*, *σ_t_*, *τ_f_*, *E*, *ν* and *c.* The iron powder content had a highly significant effect on *σ_c_*, *σ_t_*, *τ_f_* and *c*, and it had a significant effect on *ν* and *φ*. Coarse sand content had a significant effect on *σ_c_*, *E* and *c.* The multiple linear regression model has good reliability after testing, which can provide theoretical support for predicting the macroscopic mechanical parameters of rock-like materials to a certain extent. After testing, the ratio optimization program works well. When the water–paste ratio is 0.5325, the iron powder content is 3.975% and the coarse sand content is 15.967%, it is the optimal ratio of rock-like materials.

## 1. Introduction

The construction of traditional large-scale water conservancy facilities is often carried out in a complex and variable rock mechanics environment, and it is extremely important to study the mechanical properties of natural rocks for engineering carriers. However, along with the excavation of deep underground tunnels, it is difficult to sample the raw rock under the complex and variable environment of high geostress and high temperature in the deep part of the mining area. Taking the diversion tunnel project of Xinjiang Qirehatar Hydropower Station in China as an example, the maximum burial depth of the tunnel is more than 1000 m. The measured temperature of the rock wall at the palm face after blasting reached up to 119 °C, and the air temperature had exceeded 60 °C, accompanied by high-pressure 147 °C gas injection, showing typical characteristics of large burial depth and high ground temperature. Even among the same kind of rock from the same area, its internal fracture characterization and distribution characteristics show strong heterogeneity, so the results are highly discrete [1]. The use of raw rock for testing has multiple problems, such as difficult sampling, high transport costs, irregular specimens, etc., which have led many scholars to carry out experimental research through the preparation of rock-like materials instead of raw rock [2,3,4]. Rock-like materials have the advantages of low raw material costs, simple fabrication, homogeneous specimens, and easy adjustment of test conditions and protocols [5]. Stimpson [6] summarized the experience of previous scholars who used rock-like materials to replace rocks in their research, and classified common rock-like materials into two categories: non-granular [7] and granular materials. Among them, granular rock-like materials mainly include mortar materials with cement or gypsum as the cementing material. As a frictional material, mortar materials can be tested to demonstrate a high degree of similarity between their mechanical properties and those of certain rocks. By choosing appropriate proportioning factors and variable intervals for the preparation of rock-like materials and conducting mechanical property tests under macro-control, the mechanical properties can be characterized similarly to those of the original rock to a certain extent, and they can more realistically restore the damage characteristics of the rock under loading conditions [8,9,10]. Therefore, the use of rock-like materials with mechanical properties similar to those of the original rock’s characterization has a wide range of applications in mechanical test analysis [11,12,13,14].

However, how to accurately obtain rock-like materials with the most similar properties to the original rock is still a difficult problem to be solved. Previous scholars have also tried to use different mixing ratio design methods to meet various multi-objective optimization requirements [15,16,17]. Research scholars mainly seek the concrete improvement of material properties by using design of experiments (DOE) [16,17,18], statistical methods [19,20,21] and formula analysis methods [22,23]. Specific improvements in material performance are sought primarily through the use of statistical experimental designs combined with formulaic analysis. Cui et al. [5] revealed the mechanism, degree and mode of influence of the ratio of resin, hardener and accelerator as well as the freezing time on the mechanical properties of hyaline analogue specimens based on an orthogonal experimental design, and they clarified the principle of optimizing the ratio of brittle hyaline analogue specimens with different fissure structures. Gu et al. [24] used a homogeneous design to consider the effect of the doping ratio on flow properties, geometric deformation and mechanical properties; established relationship equations between factors and indicators through nonlinear regression analysis; and finally carried out multi-objective optimization to obtain the optimal material ratios. Song et al. [25] used the orthogonal test method to explore the granularity of materials with different water–cement ratios, sand–cement ratios and silica fume–cement ratios to prepare rock-like materials with similar deformation and brittleness characteristics as sandstone. Chen et al. [26] used fly ash instead of crushed gangue as a mixed filling material and used the response surface method to design 29 sets of proportion schemes to obtain the optimum proportion. Wu et al. [27] produced artificial cores using 3D printing technology and studied the condensation time, rheology, permeability and porosity of the specimens through orthogonal tests, and they optimized the material ratios to obtain the best material combinations within the range meeting the experimental requirements. Hao et al. [28] systematically analysed the effects of factors such as the water–cement ratio, glue–sand ratio, water–glass ratio, water-reducing agent, and fly ash/cement ratio on various performance indexes such as slurry flow, viscosity, setting time, urination rate, compressive strength and stone rate based on the homogeneous design method and determined the optimal mix ratio for the addition of major raw materials during grout proportioning.

To sum up, the traditional methods still have some limitations, and it is difficult to achieve accurate and efficient ratio optimization [29,30]. In particular, these methods may ignore the complexity of the hydration reaction of cement or gypsum cementitious materials, which leads to the inapplicability of mixture ratio optimization research. These limitations often appear in the analysis of key factors affecting the properties of materials, and it is impossible to realize real-time and accurate dynamic proportioning optimization [31]. In addition, the traditional method cannot meet the requirements of material properties in practical engineering applications [32]. Therefore, it is urgent to find a new method, which can surpass the existing traditional methods based on experimental design, statistics and empirical formulas. It provides an effective solution for the optimal design of the material ratio.

In recent years, with the rapid development of artificial intelligence algorithms, large data sets have been processed and analysed efficiently by simulating human thinking [33]. At the same time, the application of artificial intelligence is accompanied in the frontier fields of materials and engineering, and it shows a significant growth trend [34]. Therefore, intelligent algorithms, such as evolutionary algorithms, are introduced into material proportioning design and optimization, providing a new solution [35,36]. In particular, the genetic algorithm (GA), derived from the evolutionary algorithm, is often used to solve multi-objective problems [37]. Chen et al. [31] developed a hybrid intelligent framework based on the random forest (RF) algorithm, the least-squares support vector machine (LSSVM) algorithm and the nondominated sorting genetic algorithm with an elite strategy (NSGA-II) to realize the efficient optimization of concrete mixtures. Beji et al. [32] developed an innovative methodology to identify equivalent morphologies within a microstructure containing a circular inclusion using a coupling model based on the predictions of machine learning algorithms and the optimization of genetic algorithms. Dvoršek et al. [38] used the genetic algorithm to optimize the parameters of the Chaboche material model in an industrial environment based on experimental and simulation data.

In view of this, on the basis of the above-mentioned scholars’ research on material proportioning optimization, this study combines the traditional statistical experimental design and formula analysis with an intelligent optimization algorithm, and it carries out the genetic algorithm inversion proportioning optimization research of rock-like materials based on orthogonal experimental design. Therefore, this paper takes the rock mass (gneiss granite) in the Qire Khattar diversion tunnel project in Xinjiang, China, as the main research object. The similarity scale and dimensional analysis are used to determine the macro-mechanical parameters of the target. Based on an orthogonal experimental design, relevant indoor physical tests are carried out to obtain seven macro-mechanical parameters of rock-like materials. Based on the sensitivity variance analysis of the orthogonal test, the significant influencing factors of each ratio on mechanical parameters are judged, and the quantitative formula between the ratio factors and macro-mechanical parameters is constructed by the multiple linear regression method. Finally, the minimum value of the difference between the orthogonal test results and the theoretical value obtained by the quantitative formula is taken as the objective function, and the range of the ratio factors is taken as the constraint condition. In the MATLAB environment, an inverse matching optimization program based on genetic algorithm is written to seek the optimal matching of rock-like materials. The inversion optimization method based on the genetic algorithm proposed in this study can provide some theoretical guidance and reference significance for the research of similar materials.

## 2. Materials and Methods

### 2.1. Target Rocks and Macro-Parameters

Based on the Qire Khattar diversion tunnel project in Xinjiang, China, this study takes gneiss granite as the main research object to optimize the ratio of rock-like materials. The complexity and variability of the geology of numerous rock bodies resulted in a wide range of mechanical parameters spanning the range of the acquired raw rock. The tensile/compression ratio *σ_t_*/*σ_c_* of the target rock was taken as the main analogue in the proportion optimization, and a total of seven macro-mechanical parameters, including compressive strength *σ_c_*, tensile strength *σ_t_*, shear strength *τ_f_*, elastic modulus *E*, Poisson’s ratio *ν*, internal friction angle *φ* and cohesion *c* were considered for the study. By consulting the relevant literature, it is known that the tension–compression ratio of gneiss granite ranges from 1/8.6 to 1/18.6. In the process of tunnel excavation, we carried out a large number of sampling and mechanical tests on gneiss granite. The macroscopic parameters and tension–compression ratio of the target rock obtained from the test are shown in Table 1. According to the data obtained in Table 1, the tension–compression ratio can be obtained to be 1/15. Therefore, a tensile–compressive ratio of 1/15 for the target rock is selected as the main index to carry out the experimental study on the optimization of rock-like material ratios.

The target macroscopic parameters of the rock-like materials are co-designed based on similarity theory [39] and magnitude analysis [40]. Based on the traditional method of magnitude analysis, the static similarity physical relationship between the original rock and rock-like materials is established, and the *MLT* magnitude system with mass *M*, length *L* and time *T* as the basic magnitudes is adopted, and all other physical quantity magnitudes can be expressed by *MLT*. Since the self-weight loading of the specimen in this system cannot be achieved by applying external forces, it needs to be satisfied by the self-weight of the material itself. Therefore, before the target macro-parameters are determined, the similarity ratio of the density *ρ* of the gneiss granite to the rock-like material is taken as *S_ρ_* = 1, with *S_ρ_* as the base invariant, and, finally, the similarity ratio scale *S*_1_ is determined by a combination of the similarity theorem and the method of magnitude analysis. The rock-like material similarity scales and target macro-mechanical parameters are shown in Table 1.

### 2.2. Material and Proportioning Factors

In the design of the test, the range of values of the material proportioning factors determines the basic premise that the specimen and the target rock characterize similar mechanical properties, so the selection of variable factors and intervals ultimately determines the reliability of the test results [13]. By analysing the proportioning scheme of related rock-like materials, it can be seen that the final choice of natural river sand from Zhanghe River in Handan and the screening of coarse sand with fineness modulus of 3.1~3.7 and 300 mesh unadulterated ordinary iron powder as the aggregate can achieve the effect of improving the stability performance of the specimen and increasing the capacity of the material to a certain extent. At the same time, considering that gypsum has certain strength and brittleness characteristics due to the hydration reaction, *β*-type hemihydrate gypsum (*β*-CaSO_4_·1/2H_2_O) formed by the calcination of industrial phosphogypsum was chosen as the main cementitious material. In addition, diatomaceous earth powder [41,42] was used as a modifying material at a dosage of 1.15% of the gypsum mass. In terms of organization, diatomaceous earth itself can have good adsorption with cementitious materials such as cement or gypsum, which can provide good stability [43]. In order to ensure the smooth preparation of the specimens and strength requirements, HG High Efficiency Gypsum Retarder with 0.05% gypsum mass was also added. Since diatomaceous earth and gypsum retarder are used as external admixtures, their percentage of admixture is determined by the quality of the gypsum, so they are used as invariant factors in this experimental design.

Based on the analysis of the results of a large number of preliminary pre-tests, water mass/gypsum mass (water–plaster ratio), iron powder mass/gypsum mass × 100% (iron powder content), and coarse sand mass/gypsum mass × 100% (coarse sand content) were finally selected as the three proportionality factors for this study. The study intervals for each proportioning factor were determined separately and are shown in Table 2. The specimens prepared using this proportioning method are relatively ideal highly brittle rock-like materials, with a view to carrying out subsequent experimental studies on optimizing the proportioning of rock-like materials through different combinations of proportions.

### 2.3. Sample Preparation and Experimental Design

Based on the selected seven macro-mechanical parameters, it can be seen that the mechanical performance test includes a total of four tests, uniaxial compression, Brazilian splitting, direct shear and triaxial compression, so *Ф*50 × 100 mm (diameter × height) cylindrical moulds and 100 × 100 × 100 mm (length × width × height) square moulds are used.

**Figure 1:**
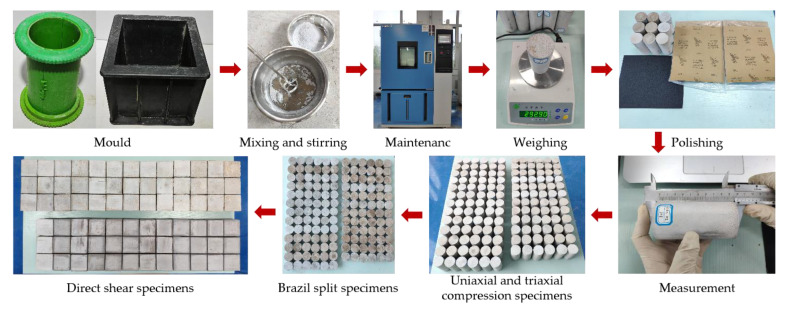
Preparation process of rock-like samples.

The specimens were prepared by weighing phosphogypsum, diatomite, gypsum retarder and the mass of water, iron powder and coarse sand corresponding to the water–paste ratio of each group of tests. Phosphogypsum, diatomite and gypsum retarder were mixed evenly and poured into water. They were stirred for 20 s, and then the proportioning factors were poured into the mixing slurry and mixed thoroughly. The gypsum mix was stirred for 1 min, used to fill both moulds, vibrated for 2 min and then demoulded after 2 h of resting. The demoulded specimen was put into the high- and low-temperature alternating humidity and heat experimental chamber. The temperature was maintained at 40° and humidity at 0 conditions of maintenance. The specimen mass was weighed regularly until it reached a constant value. The finished specimen was taken out and all surfaces of the specimen were polished with 60-grit and 220-grit sandpaper in turn. The size of the polished sample was measured with a vernier calliper. The preparation process of rock-like samples is shown in Figure 1.

**Figure 2:**
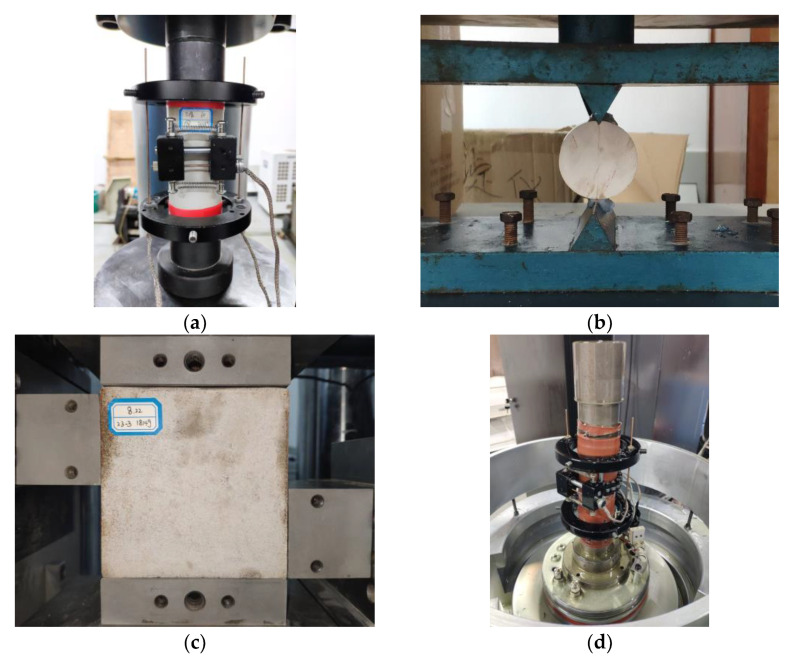
Mechanical property tests: (**a**) uniaxial compression; (**b**) Brazilian splitting; (**c**) triaxial compression; (**d**) direct shear.

The compressive strength *σ_c_*, modulus of elasticity *E* and Poisson’s ratio *ν* of the rock-like material specimens can be obtained by a uniaxial compression test in the TAW-2000 electro-hydraulic servo rock triaxial tester produced by Changchun Chaoyang Test Instrument Co., Ltd., Changchun, China, as shown in Figure 2a. The tensile strength *σ_t_* can be obtained from the Brazilian splitting test in a microcomputer-controlled electronic universal testing machine with a fixed mould, as shown in Figure 2b. The shear strength *τ_f_* can be obtained by a direct shear test with the ZTRS-210 large-tonnage rock straight shear instrument produced by Changchun Chaoyang Test Instrument Co., Ltd., Changchun, China, as shown in Figure 2c. The angle of internal friction *φ* and cohesion *c* were obtained based on triaxial compression tests and according to the Mohr–Coulomb damage criterium by plotting the Mohr stress circle and strength envelope, as shown in Figure 2d. The four mechanical properties were tested using displacement-controlled loading, and the loading rate was kept at 0.02 mm/min, as shown in Figure 2. At the same time, in order to minimize the dispersion of the test results, not less than three parallel specimens were used for each set of condition tests.

**Figure 3:**
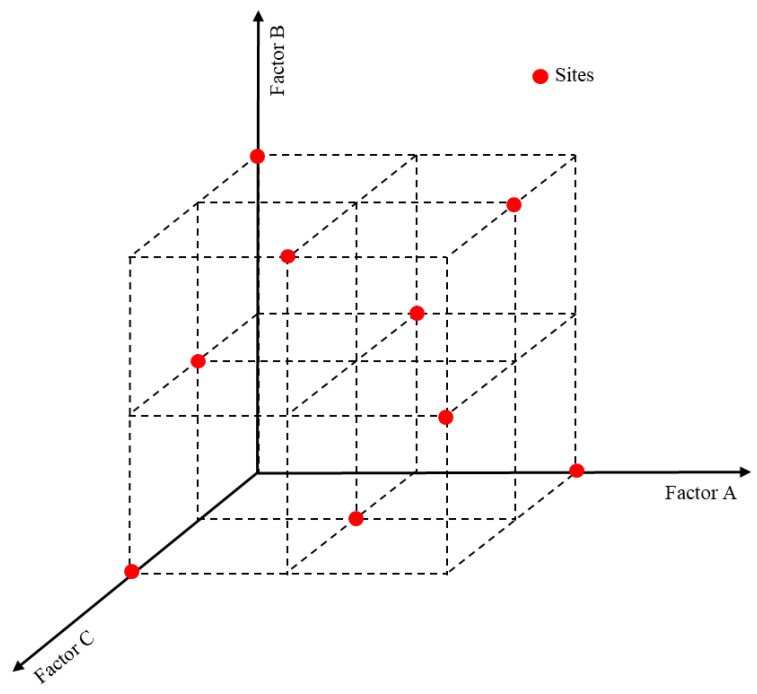
Experimental point distribution of orthogonal experimental design.

Statistical experiment design can provide scientific and reasonable methods to arrange experiments, resulting in more reliable results and conclusions with less time and financial cost [44]. Orthogonal experimental design [45], based on probability theory, mathematical statistics and practical experience, which are used together to establish a standardized orthogonal table to arrange the process of experimental factors and level combinations for multi-factor and multi-level optimization problems with simple, efficient and other advantages, is shown in Figure 3. In this experiment, based on the orthogonal test method, the rock-like material *σ_c_*, *σ_t_*, *τ_f_*, *E*, *ν*, *φ* and *c* were selected as the indexes for investigation, and the rock-like material proportioning variables A (water–paste ratio), B (iron powder content) and C (coarse sand content) were the influencing factors. Five factor levels were set for each factor, and an orthogonal test factor level design of 3 factors and 5 levels was obtained, as shown in Table 3.

## 3. Results and Analyses

### 3.1. Orthogonal Test Results

Based on the orthogonal test factor level design in Table 3, the L_25_(5^3^) orthogonal test programme was completed by SPSS 26 software, and the final orthogonal test results and 25 groups of macro-mechanical parameters are shown in Table 4.

### 3.2. Sensitivity ANOVA

The sensitivity analysis of variance (ANOVA) based on the orthogonal test can not only judge the sensitivity of each factor to the inspection index but also accurately express the quantitative relationship between them. In this experiment, α > 0.05 is selected as an unsignificant statistical difference and no sign is counted. When 0.01 < α ≤ 0.05, signified by ‘(*)’, it indicates a statistically significant difference. When 0.001 < α ≤ 0.01, ‘*’ indicates a highly significant statistical difference. α ≤ 0.001 is represented by ‘**’ and indicates an extremely statistically significant difference. The above symbols are used to distinguish the significant influence of the three ratio factors on the seven macroscopic mechanical parameters, as shown in Table 5.

### 3.3. Multiple Linear Regression Analysis

#### 3.3.1. Model Construction

In order to further investigate the relationship between the proportioning factors and the macroscopic mechanical parameters, the test results were fitted and analysed using multiple linear regression. The reliability of the indoor physical test results can also be checked by constructing multiple linear regression equations to compare and analyse the fitted values with the test values.

Assume the following equation for the multiple linear regression model independent variable *x_i_* and dependent variable *y*:

(1) $$\hat{y}=a_{0}+a_{1}x_{1}+a_{2}x_{2}+\cdots +a_{m}x_{m}$$

where *a*_0_, *a*_1_, ···, *a_m_* is the coefficient of the regression model, and the test result *y_i_* (*i* = 1, 2, ···, *n*) is put back into Equation (1), so that the residual sum of squares can be expressed as follows:

(2) $$Q=\sum_{i=1}^{n}{\left(y_{i}-{\hat{y}}_{i}\right)}^{2}=\sum_{i=1}^{n}{\left(y_{i}-a_{0}-a_{1}x_{1}-a_{2}x_{2}-\cdots -a_{m}x_{m}\right)}^{2}$$

Based on the least square method, when *Q* is minimized, Equation (2) should satisfy the following relationship:

(3) $$\frac{\partial Q}{\partial a_{j}}=0;j=1,2,\cdots ,m$$

By calculating Equation (3), the resultant output is the following system of linear equations:

(4) $$\left\{\begin{array}{l} na_{0}+a_{1}\sum_{i=1}^{n}x_{1i}+a_{2}\sum_{i=1}^{n}x_{2i}+\cdots +a_{m}\sum_{i=1}^{n}x_{mi}=\sum_{i=1}^{n}y_{i}a_{0}\sum_{i=1}^{n}x_{1i}+a_{1}\sum_{i=1}^{n}x_{1i}^{2}+a_{2}\sum_{i=1}^{n}x_{1i}x_{2i}+\cdots +\\ a_{m}\sum_{i=1}^{n}x_{1i}x_{mi}=\sum_{i=1}^{n}x_{1i}y_{i}a_{0}\sum_{i=1}^{n}x_{mi}+a_{1}\sum_{i=1}^{n}x_{1i}x_{mi}+a_{2}\sum_{i=1}^{n}x_{2i}x_{mi}+\cdots +a_{m}\sum_{i=1}^{n}x_{mi}^{2}=\sum_{i=1}^{n}x_{mi}y_{i} \end{array}\right.$$

Equivalent substitution of the system of linear equations in Equation (4) is performed, and the relations are expressed in Equations (5) to (8):

(5) $${\bar{x}}_{j}=\frac{1}{n}\sum_{i=1}^{n}x_{ij}$$

(6) $$\bar{y}=\frac{1}{n}\sum_{i=1}^{n}y_{ij}$$

(7) $$L_{jk}=L_{kj}=\sum_{i=1}^{n}\left(x_{ji}-{\bar{x}}_{j}\right)(x_{ki}-{\bar{x}}_{k})=(\sum_{i=1}^{n}x_{ji}x_{ki})-n{\bar{x}}_{j}{\bar{x}}_{k};k=1,2,\cdots ,m$$

(8) $$L_{jy}=\sum_{i=1}^{n}\left(x_{ji}-{\bar{x}}_{j}\right)(y_{i}-\bar{y})=(\sum_{i=1}^{n}x_{ji}y_{i})-n{\bar{x}}_{j}\bar{y};k=1,2,\cdots ,m$$

Then, Equation (4) finally reduces to the following:

(9) $$\left\{\begin{array}{l} a_{0}=\bar{y}-a_{1}{\bar{x}}_{1}-a_{2}{\bar{x}}_{2}-\cdots -a_{m}{\bar{x}}_{m}\\ L_{11}a_{1}+L_{12}a_{2}+\cdots +L_{1m}a_{m}=L_{1y}\\ \cdots \\ L_{m1}a_{1}+L_{m2}a_{2}+\cdots +L_{mm}a_{m}=L_{my} \end{array}\right.$$

Solving the final simplified system of linear Equation (9) yields the regression model coefficients *a*_0_, *a*_1_, ···, *a_m_*, and the final multiple linear regression model is obtained by substituting it back into Equation (1). Combined with the results of ANOVA in Table 5, the effect of significant factors should be prioritized in the linear regression analysis, thus determining the construction of a regression model to satisfy rock-like material properties *σ_c_*, *σ_t_*, *τ_f_*, *E*, *ν*, *φ* and *c* as follows:

(10) $$\left\{\begin{array}{l} y_{\sigma_{c}}=a_{\sigma_{c0}}+a_{\sigma_{c1}}x_{1}+a_{\sigma_{c2}}x_{2}+a_{\sigma_{c3}}x_{3}\\ y_{\sigma_{t}}=a_{\sigma_{t0}}+a_{\sigma_{t1}}x_{1}+a_{\sigma_{t2}}x_{2}\\ y_{\tau_{f}}=a_{\tau_{f_{0}}}+a_{\tau_{f1}}x_{1}+a_{\tau_{f2}}x_{2}\\ y_{E}=a_{E_{0}}+a_{E_{1}}x_{1}+a_{E_{3}}x_{3}\\ y_{\nu }=a_{\nu_{0}}+a_{\nu_{1}}x_{1}+a_{\nu_{2}}x_{2}\\ y_{\varphi }=a_{\varphi_{0}}+a_{\varphi_{2}}x_{2}\\ y_{c}=a_{c_{0}}+a_{c_{1}}x_{1}+a_{c_{2}}x_{2}+a_{c_{3}}x_{3} \end{array}\right.$$

where *a_ij_* (*i* = *σ_c_*, *σ_t_*, *τ_f_*, *E*, *ν*, *φ*, *c*) is the model regression coefficient, *x*_1_ is the water–paste ratio, *x*_2_ (%) is the iron powder content and *x*_3_ (%) is the coarse sand content.

#### 3.3.2. Conditional Decision

Before the construction of a multiple linear regression model, the linear relationship, variable independence, residual normality and residual variance homogeneity of the regression model should be determined first.

**Figure 4:**
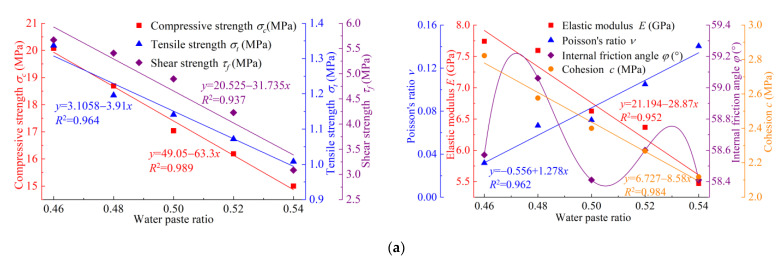

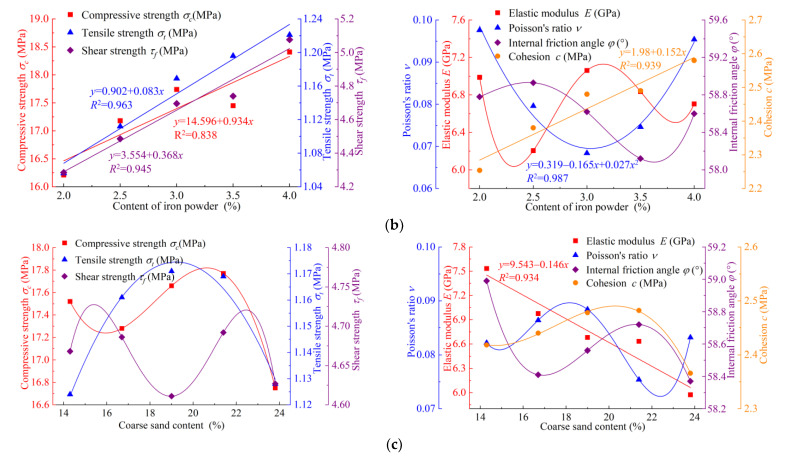
The relationship between various factors and macroscopic mechanical parameters: (**a**) water–paste ratio; (**b**) content of iron powder; (**c**) coarse sand content.

Firstly, according to the orthogonal test results in Table 4, the linear relationship between each factor and macro-mechanical parameter can be judged, and at the same time, the influence law of each ratio factor on macro-mechanical parameters can be analysed more intuitively, as shown in Figure 4.

As can be seen from Figure 4, all the macro-mechanical parameters except *φ* meet the linear relationship. Among them, *σ_c_*, *σ_t_*, *τ_f_*, *E* and *c* are linearly related to the water–gypsum ratio and decrease with the increase in the water–gypsum ratio, which can adjust the mechanical parameters of the sample to a great extent. There is a positive linear relationship between *ν* and the water–gypsum ratio, and it increases with the increase in the water–gypsum ratio. Except for *φ*, the other six macroscopic mechanical parameters showed a high degree of linearity with the water–paste ratio, which is consistent with the results of variance analysis (Figure 4a). The nonlinear relationship of *φ* is mainly caused by the joint influence of particle gradation and shape, mineral composition, density, water content, particle size, particle surface roughness, compactness and void ratio. Many factors jointly affect the *φ* of materials and then affect their shear strength and stability. Based on *β*-gypsum hemihydrate (*β*-CaSO_4_·1/2H_2_O) as the main cementing material, this study carried out the optimization of rock-like materials. Because there are differences in the properties of rock-like materials with rock and gypsum as the main body, the influencing factors, such as particle size distribution and shape, mineral composition and the density of the two materials, are significantly different, which leads to the inability to achieve better linear correlation between *φ* and ratio factors.

*σ_c_*, *σ_t_*, *τ_f_* and *c* have a positive linear relationship with the content of iron powder. This is because iron powder, as aggregate particles, with the increase in iron powder content, the contact area with gypsum, a cementing material, is increased, so that the cementation between particles is enhanced and the compactness of the internal structure of the material is improved, which has a significant impact on *σ_c_*, *σ_t_*, *τ_f_* and *c* (Figure 4b). The coarse sand content only has a negative linear relationship with *E*, but it has no linear relationship with the other mechanical parameters. It can also be verified that the influence degree of coarse sand content on various macro-mechanical parameters in Table 5 is not significant compared with the above two proportioning factors (Figure 4c).

The collinearity diagnosis results of the regression model are shown in Table 6, and the inspection indexes are that the characteristic roots of each dimension of the seven macro-mechanical parameters are all greater than 0 and the conditional indexes are all less than 10, which can satisfy the independence of variables. As can be seen from Table 6, the three proportioning factors are independent of each other and there is no multicollinearity.

**Figure 5:**
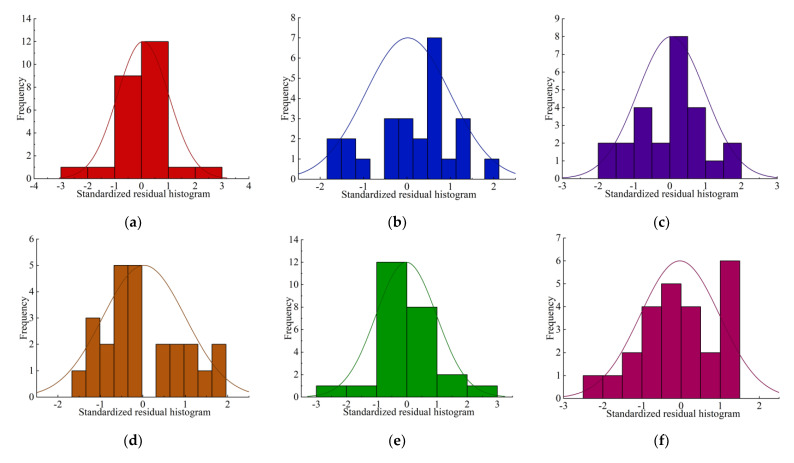

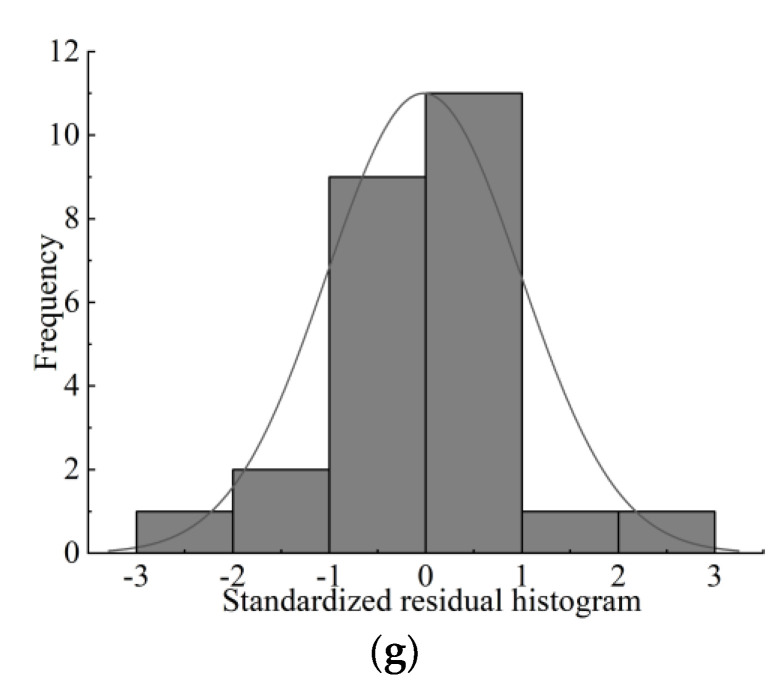
Regression model standardized residual histograms: (**a**) compressive strength *σ_c_*; (**b**) tensile strength *σ_t_*; (**c**) shear strength *τ_f_*; (**d**) elastic modulus *E*; (**e**) Poisson’s ratio *ν*; (**f**) internal friction angle *φ*; (**g**) cohesion *c.*

Figure 5 shows the standardized residual histogram of the regression model. It can be seen that the standardized residual values of the regression value and the test value are roughly in accordance with the normal distribution. The residual normality is satisfied.

**Figure 6:**
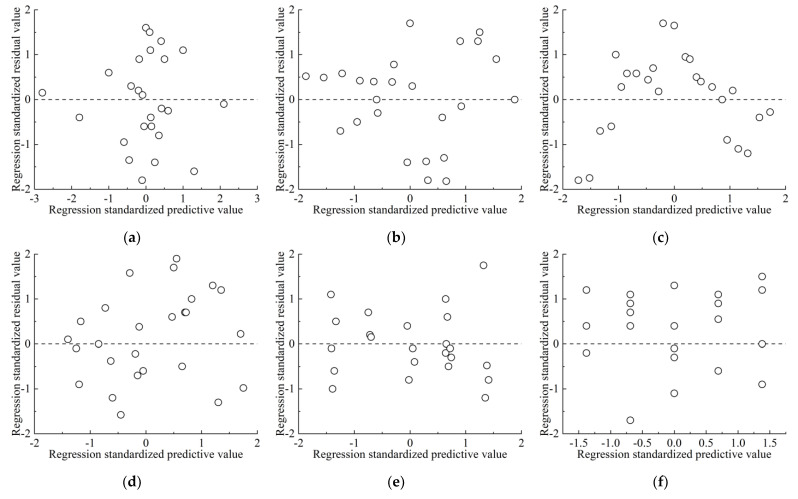

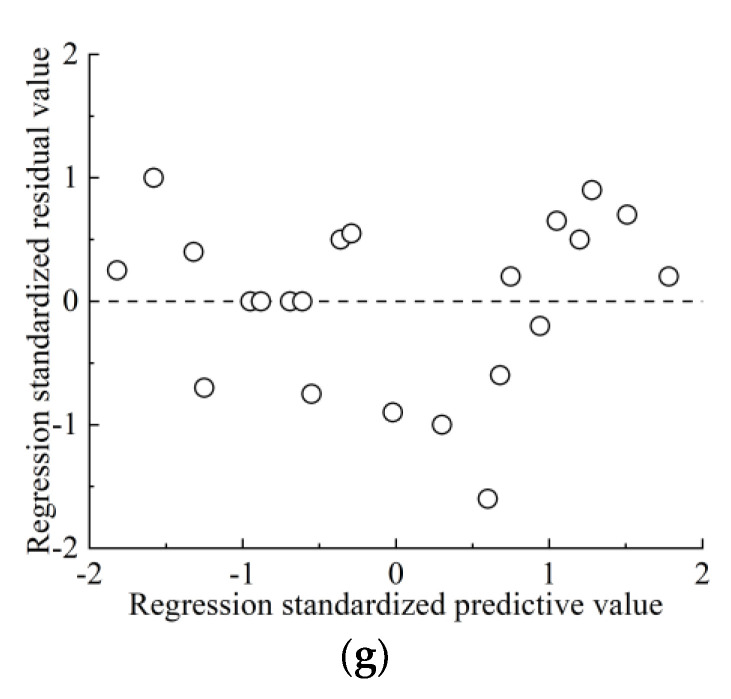
Standardized residual scatter plots of regression model: (**a**) compressive strength *σ_c_*; (**b**) tensile strength *σ_t_*; (**c**) shear strength *τ_f_*; (**d**) elastic modulus *E*; (**e**) Poisson’s ratio *ν*; (**f**) internal friction angle *φ*; (**g**) cohesion *c.*

Figure 6 shows the standardized residual scatter plot of each regression model, in which the residuals of each mechanical parameter are evenly dispersed on both sides of 0, and no obvious regularity is found, indicating that the standard residual results meet the homogeneity of variance.

In summary, the constructed *σ_c_*, *σ_t_*, *τ_f_*, *E*, *ν* and *c* regression models all meet the preconditions of multiple linear regression analysis. However, as can be seen from Figure 4, *φ* has no linear relationship with the water–gypsum ratio, iron powder content and coarse sand content, which cannot meet the prerequisite of multiple linear regression analysis. Therefore, *φ* is not considered in the subsequent multiple linear regression analysis.

#### 3.3.3. Analysis and Inspection

The results of the orthogonal test in Table 4 can be obtained by multiple linear regression analysis with Origin 2021 software. The multiple linear regression equations of various macro-mechanical parameters of rock-like materials are shown in Table 7. As can be seen from Table 7, the regression equations of *σ_c_*, *σ_t_*, *τ_f_* and *c* all have coefficients *R*^2^ above 0.9. The coefficient *R*^2^ determined by the regression equation of *E* and *ν* is between 0.7 and 0.85, which is lower than the above four macro-mechanical parameters. Therefore, within the range of considering the error influence of certain non-experimental factors, the regression equations of all macro-mechanical parameters can be in good agreement with the orthogonal test results; that is, the correlation between different ratio factors and horizontal combinations and macro-mechanical parameters can be effectively reflected.

**Figure 7:**
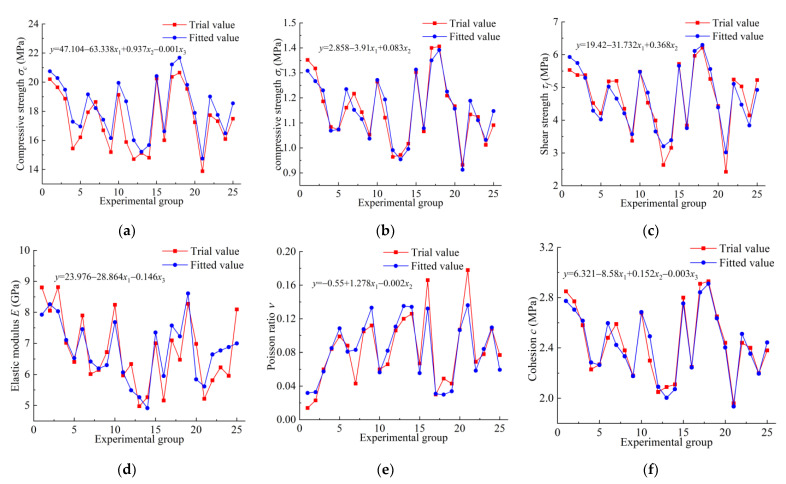
Comparison of regression equation fitting values and experimental values: (**a**) compressive strength *σ_c_*; (**b**) tensile strength *σ_t_*; (**c**) shear strength *τ_f_*; (**d**) elastic modulus *E*; (**e**) Poisson’s ratio *ν*; (**f**) cohesion *c.*

The fitting value $y_{i}^{*}$ of *σ_c_*, *σ_t_*, *τ_f_*, *E*, *ν* and *c* can be obtained by bringing the orthogonal test results into the regression model in Equation (12). Compare it with the test values of mechanical properties in Table 4, as shown in Figure 7. In Figure 7, the *x*-axis is 25 experimental groups based on orthogonal design, in which each ratio combination has three ratio factors. The *y*-axis is the corresponding macro-mechanical parameters *σ_c_*, *σ_t_*, *τ_f_*, *E*, *ν* and *c*. Taking the similarity between the curve of the regression equation and the curve of the test results as the standard criterium, the fitting effect of the regression equation is analysed, and the reliability of test results is tested.

As can be seen from Figure 7, the regression equation curves of *σ_c_*, *σ_t_*, *τ_f_*, *ν* and *c* are almost similar to the experimental results, in which *σ_c_*, *σ_t_*, *τ_f_* and *c* are almost completely coincident. The curve coincidence degree of *E* is slightly lower, but the curve trend is basically the same.

**Figure 8:**
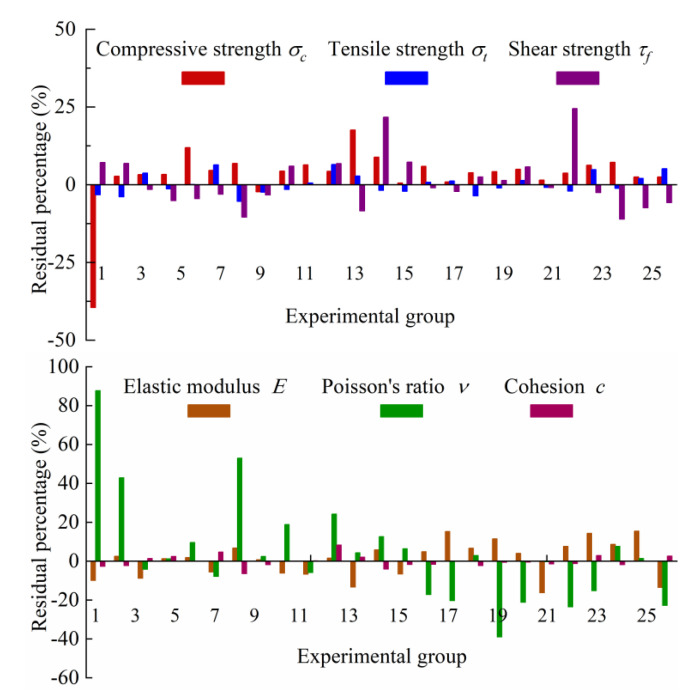
Residual percentage of regression model.

In order to further test the reliability of the regression model, the dimensionless number $\left|y_{i}^{lab}-y_{i}^{*}\right|/y_{i}^{lab}$ is used to investigate the error of the regression model. The residual percentage calculated according to Formula (11) is shown in Figure 8.

(11) $$\frac{\left|y_{i}^{lab}-y_{i}^{*}\right|}{y_{i}^{lab}}\times 100\%$$

As can be seen from Figure 8, except for test groups 1, 5 and 12, the percentage of the residual difference of *σ_c_* regression results is less than 10%. The residual values of all experimental groups of *σ_t_* and *c* are less than 10%. For *τ_f_*, except for test groups 13 and 21, the residual values of other results are less than 10%. In terms of *E,* except for the 16th, 20th and 24th experimental groups, the residual values of other results are less than 15%. For *ν,* except for the test groups 1, 2, 7, 11 and 18, the residual values of other results are less than 15%. Because the model is based on multiple linear regression theory, it mainly considers the significant influence of linear factors. The proportional variables of the nonlinear relationship are not included in the scope of the equation, which will lead to some errors. However, the error of the model is small and the accuracy meets the test requirements of rock-like materials, so the model can be used to predict the macro-mechanical parameters of rock-like materials. At the same time, the quantitative relationship between proportioning factors and mechanical parameters is constructed based on the regression equation, which lays a foundation for further quantitative research on the proportioning optimization of rock-like materials.

## 4. Optimization Model for Rock-Like Material Ratios

### 4.1. Optimization Models and Algorithms

**Figure 9:**
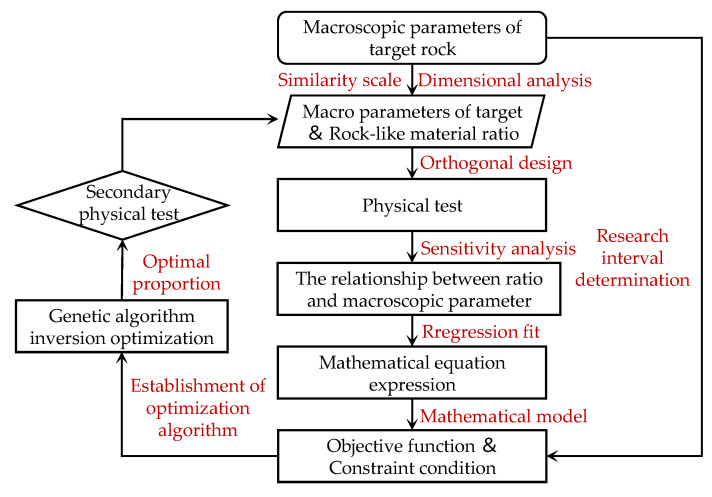
Inversion ratio optimization program based on genetic algorithm.

In many practical projects, the general way to solve the optimization problem is to choose the factors (independent variables) that need to be considered in the solved problem. Under a series of related constraints (constraints), the design index (objective function) finally reaches the optimal solution. Usually, optimization problems can be expressed in the form of mathematical programming, which can be transformed into mathematical problems in engineering optimization design, and mathematical models of optimization-related problems can be designed to obtain the optimal solution. The optimization model of rock-like material ratios is constructed according to the objective function and constraint conditions of the research object, and finally the optimal matching ratio of rock-like material is deduced. The genetic algorithm is used to invert the proportioning optimization program, as shown in Figure 9.

Seeking the optimal proportion of rock-like materials is to restore the macro-mechanical properties of the target original rock to the greatest extent. We take the multiple linear regression equation in Table 7 as the quantitative formula between the rock-like material ratio and the macro-mechanical parameters. Assuming the quantitative relationship between them, the calculated fitting value is $y_{i}^{*}(i=1,2,\cdots ,n)$. The experimental values of 25 groups of macroscopic mechanical parameters are $y_{i}^{lab}(i=1,2,\cdots ,n)$, where n is the number of macroscopic mechanical parameters studied. The optimization problem of the rock-like material ratio is transformed into finding the most suitable combination of rock-like material ratio factors *X*_1_, *X*_2_, ···, *X_k_*, where k is the number of rock-like material ratio factors, and the difference between $y_{i}^{*}$ and $y_{i}^{lab}$ should be as small as possible. At the same time, considering the magnitude difference among the seven macro-mechanical parameters studied, a dimensionless number can be used to define the relative deviation $\left|y_{i}^{lab}-y_{i}^{*}\right|/y_{i}^{lab}$ to describe the difference between $y_{i}^{*}$ and $y_{i}^{lab}$. The optimization problem of rock-like material proportion in this design can now be transformed into finding the best combination of proportioning factors *X*_1_, *X*_2_, ···, *X_k_*, so that $\left|y_{i}^{lab}-y_{i}^{*}\right|/y_{i}^{lab}$ can reach the minimum value. At this time, *X*_1_, *X*_2_, ···, *X_k_* is the optimal ratio.

In the process of searching for the best combination of proportioning factors *X*_1_, *X*_2_, ···, *X_k_*, it is necessary to determine the range of proportioning factors of rock-like materials, and set it to [*a_j_*,*b_j_*] (*j* = 1, 2, ···, *k*), so that the general form of inversion based on the genetic algorithm can be obtained as shown in Formula (12):

Objective function:

(12) $$\min f(x)=\sum_{i=1}^{n}\frac{\left|y_{i}^{lab}-y_{i}^{*}\right|}{y_{i}^{lab}}$$

Constraint condition:

(13) $$a_{j}\leq X_{j}\leq b_{j}(1\leq j\leq k)$$

According to the analysis of the above test results and the above assumptions, the fitting values of macro-mechanical parameters are determined as follows: $\sigma_{c}^{*}$, $\sigma_{t}^{*}$, $\tau_{f}^{*}$, $E^{*}$, $\nu^{*}$ and $c^{*}$. The experimental values of 25 groups of macro-mechanical parameters designed by the orthogonal test are set as follows: $\sigma_{c}^{lab}$, $\sigma_{t}^{lab}$, $\tau_{f}^{lab}$, $E^{lab}$, $\nu^{lab}$ and $c^{lab}$. The water–gypsum ratio, iron powder content and coarse sand content are taken as independent variables in the inversion optimization model based on the genetic algorithm. Let the water–gypsum ratio, iron powder content and coarse sand content be *X*_1_, *X*_2_, *X*_3_, respectively. The ratio optimization model in Formula (14) based on genetic algorithm inversion can be obtained.

Objective function:

(14) $$\min f(x)=\frac{\left|\sigma_{c}^{lab}-\sigma_{c}^{*}\right|}{\sigma_{c}^{lab}}+\frac{\left|\sigma_{t}^{lab}-\sigma_{t}^{*}\right|}{\sigma_{t}^{lab}}+\frac{\left|\tau_{f}^{lab}-\tau_{f}^{*}\right|}{\tau_{f}^{lab}}+\frac{\left|E^{lab}-E^{*}\right|}{E^{lab}}+\frac{\left|\nu^{lab}-\nu^{*}\right|}{\nu^{lab}}+\frac{\left|c^{lab}-c^{*}\right|}{c^{lab}}$$

Constraint condition:

(15) $$\left\{\begin{array}{l} 0.46\leq X_{1}\leq 0.48\\ 2\%\leq X_{2}\leq 4\%\\ 14.3\%\leq X_{3}\leq 23.8\% \end{array}\right.$$

The essence of the optimization algorithm is the calculation method of obtaining the target information from the mathematical model, so choosing the appropriate optimization algorithm can achieve the ideal result. Traditional optimization algorithms perform well in simple optimization problems such as continuity and linearity, such as the interior point method and sequential quadratic programming method. However, their limitations are also extremely obvious, such as a strong dependence on the initial value, more requirements on the form and solution space of the objective function, and most of them take continuity and derivation as the limiting criteria. Therefore, in the process of solving many complex problems, there will be cases where it is impossible or only local optimal solutions are obtained. Modern intelligent optimization algorithms, such as the genetic algorithm, ant colony algorithm, tabu search algorithm and particle swarm optimization algorithm, can make up for the shortcomings of traditional optimization algorithms to a great extent. Based on the consideration of an objective function and constraint conditions in this optimization model design, a mature global search algorithm–genetic algorithm is finally selected as the optimization algorithm of Formula (14).

### 4.2. Realization of Proportion Optimization Program

The program design of inverse proportion optimization based on the genetic algorithm is carried out in the MATLAB environment, and the program design is carried out in the form of a command call in the genetic algorithm toolbox.

The definition of fitness function is generally related to solving problems. In the genetic algorithm toolbox, the default solution is to find the minimum value of the fitness function. The inversion principle of the algorithm is that the smaller the fitness value of an individual, the closer it is to the optimal solution. The fitness function for finding the optimal proportion of rock-like materials is the same as the objective function in Formula (14), and it can be expressed as Formula (16):

(16) $$F\mathrm{it}(x)=\frac{\left|\sigma_{c}^{lab}-\sigma_{c}^{*}\right|}{\sigma_{c}^{lab}}+\frac{\left|\sigma_{t}^{lab}-\sigma_{t}^{*}\right|}{\sigma_{t}^{lab}}+\frac{\left|\tau_{f}^{lab}-\tau_{f}^{*}\right|}{\tau_{f}^{lab}}+\frac{\left|E^{lab}-E^{*}\right|}{E^{lab}}+\frac{\left|\nu^{lab}-\nu^{*}\right|}{\nu^{lab}}+\frac{\left|c^{lab}-c^{*}\right|}{c^{lab}}$$

where Fit(x) is the fitness value. $\sigma_{c}^{lab}$ (compressive strength), $\sigma_{t}^{lab}$ (tensile strength), $\tau_{f}^{lab}$ (shear strength), $E^{lab}$ (elastic modulus), $\nu^{lab}$ (Poisson’s ratio) and $c^{lab}$ (cohesion) are all calculated by indoor physical experiments, while $\sigma_{c}^{*}$ (compressive strength), $\sigma_{t}^{*}$ (tensile strength), $\tau_{f}^{*}$ (shear strength), $E^{*}$ (elastic modulus), $\nu^{*}$ (Poisson’s ratio) and $c^{*}$ (cohesion) are all calculated by multiple linear regression equations as shown in Table 7.

Constraints in the genetic algorithm toolbox are divided into three types, namely equality constraints, inequality constraints and boundary constraints. According to the constraint conditions of the proportioning factors in the optimized proportioning model based on the genetic algorithm in Formula (15), at present, only the boundary constraints in the genetic algorithm are set to limit the starting and ending values of the three proportioning factors of rock-like materials, as shown in Formula (17):

Constraint condition:

(17) $$\left\{\begin{array}{l} 0.46\leq X_{1}\leq 0.48\\ 2\%\leq X_{2}\leq 4\%\\ 14.3\%\leq X_{3}\leq 23.8\% \end{array}\right.$$

In comparison to the traditional search algorithm, the genetic algorithm searches globally from a group of randomly generated initial solutions, which are called a ‘population’. Each individual in a population is called a ‘chromosome’. These ‘chromosomes’ continue to evolve in the subsequent process known as ‘heredity’. As the core concept of the genetic algorithm, ‘natural selection and survival of the fittest’, evolution is the process of iterative optimization.

**Figure 10:**
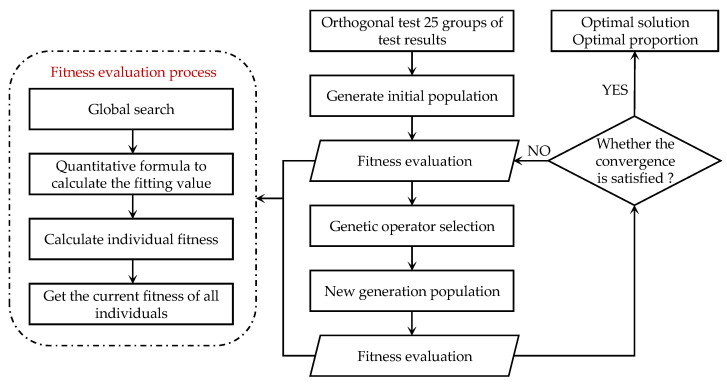
Inversion of proportion optimization process based on genetic algorithm.

Based on the concept of the genetic algorithm, the optimization program execution flow of the genetic algorithm inversion ratio optimization model shown in Formula (14) is briefly described, as shown in Figure 10.

1. Produce an initial population {*x*}^0^, and *N* individuals are randomly generated (taken as 100 in this paper).
2. Evaluate the fitness of each individual in the group:
  ① Assume that *k* = 1.
  ② Judge the interval of each microscopic parameter in the *kth* individual {*x_k_*}^0^.
  ③ Select the corresponding quantitative relationship to calculate the value of $\sigma_{c}^{*}$, $\sigma_{t}^{*}$, $\tau_{f}^{*}$, $E^{*}$, $\nu^{*}$ and $c^{*}$.
  ④ Calculate the fitness value of individual {*x_k_*}^0^ according to Equation (15).
  ⑤ *k* = *k* + 1.
  ⑥ If *k* ≤ *N*, continue the calculation. If *k* > *N*, exit the calculation as it is complete.
3. Select excellent individuals and deposit them in the mating pool.
4. Use the crossover and mutation operators to form the population {*x*}*^i^* for the next generation in the mating pool.
5. Repeat the steps in (2) to evaluate the fitness of individuals in the new population.
6. If the end condition is met, stop and obtain the optimal solution; otherwise, go to step (3) to continue the calculation.

### 4.3. Test of Inversion Optimization Effect

Substitute the target macro-mechanical parameter values of rock-like materials in Table 1 into the genetic algorithm inversion matching optimization program, and calculate the matching factor content of rock-like materials in Table 8.

**Figure 11:**
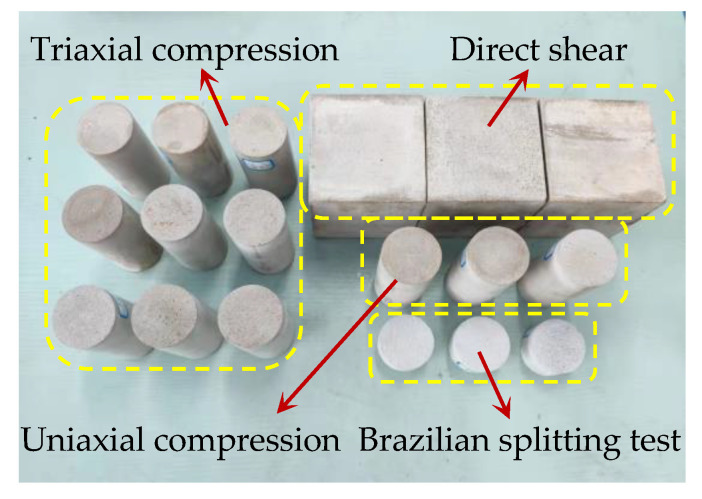
Preparation of samples by secondary indoor physical test.

Using the optimization calculation results in Table 8, a second indoor physical experiment was carried out to check the inversion results and optimization accuracy. In order to avoid human factors interfering with the inspection process, it should be consistent with the sample preparation and mechanical property test methods in Section 2.3. At the same time, in order to reduce the discreteness of the test results, no less than three samples should be tested under each working condition. The calculation of the *c* value needs to be verified by a triaxial compression test, and three samples are prepared for each confining pressure. Considering the existence of non-test error factors, repeated tests are carried out on the optimized results to ensure the effectiveness of the verification test. The samples required for the secondary indoor physical test are shown in Figure 11.

**Figure 12:**
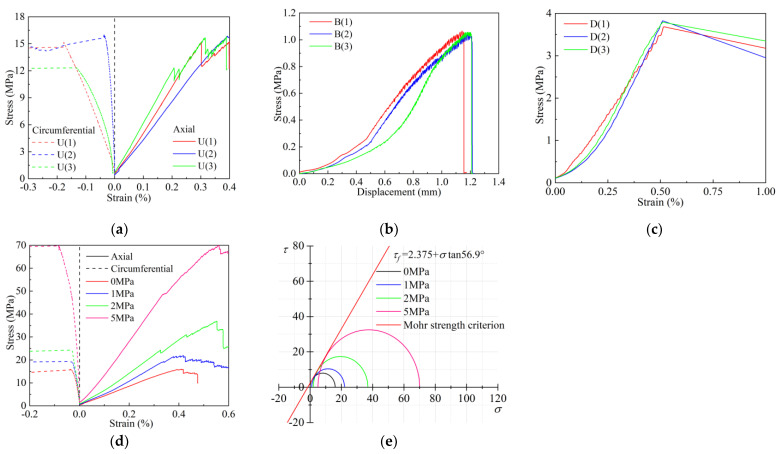
Secondary indoor physical tests: (**a**) uniaxial compression test; (**b**) Brazilian splitting test; (**c**) direct shear test; (**d**) triaxial compression test; (**e**) Mohr stress circle and strength envelope line.

*σ_c_*, *E* and *ν* can be obtained by a uniaxial compression test. The stress–strain curve of the uniaxial compression test is shown in Figure 12a. A Brazilian splitting test can obtain *σ_t_*. The stress–displacement curve of the Brazilian splitting test is shown in Figure 12b. *τ_f_* can be obtained by a direct shear test. The stress–strain curve of the direct shear test is shown in Figure 12c. According to the Mohr–Coulomb failure criterium, *c* can be obtained by a triaxial compression test. The stress–strain curves of the triaxial compression test under different confining pressures are shown in Figure 12d. The Mohr stress circle and Mohr strength envelope of the triaxial compression test are shown in Figure 12e.

Based on the mean, standard deviation, coefficient of variation and relative error, the results of the secondary indoor physical tests were tested. Since *c* is obtained by drawing the Mohr stress circle and the Mohr strength envelope from the mean values of the three samples under each confining pressure, *σ_t_*/*σ_c_* is obtained by the ratio of the mean values of *σ_t_* and *σ_c_*. Therefore, only one set of *c* and *σ_t_*/*σ_c_* is obtained, and there is no standard deviation and coefficient of variation.

The calculation formula of relative error is shown in Formula (18):

(18) $$y=\frac{\left|x^{avg}-x^{tar}\right|}{x^{avg}}\times 100\%$$

where *x* is the independent variable (*σ_c_*, *σ_t_*, *τ_f_*, *E*, *ν* and *c*), *y* is the relative error, *x^avg^* is the average value of macro-parameters in the second indoor physical test and *x^tar^* is the target macro-parameter value.

The results of inspection are shown in Table 9. It can be seen from Table 9 that the standard deviation and coefficient of variation of various macro-mechanical parameters are kept below 0.6 and 1.25, respectively, indicating that the dispersion degree of the test results is not great and the stability is good. The relative errors of various macro-mechanical parameters are all below 3.5%. Especially, the relative error of the main rock-like reference index *σ_t_*/*σ_c_* can reach the minimum error value of 0.107%, and good optimization results have been achieved. To sum up, under the premise of considering the influence of non-experimental factors, the test shows that the effect and accuracy of the genetic algorithm inversion ratio optimization program are good, which has certain reference value.

## 5. Conclusions

In this paper, gneiss granite was taken as the target analogy rock. Firstly, the target macroscopic parameters of rock-like materials were obtained using a similarity scale and dimensional analysis. Based on orthogonal experimental design and sensitivity variance analysis, the significant factors influencing mechanical parameters were explored. By constructing multiple linear regression equations, the quantitative relationship between proportioning factors and macro-mechanical parameters was obtained. Finally, an inverse proportion optimization program based on the genetic algorithm was written in the MATLAB environment. The main conclusions of this study are as follows.

(1) According to the sensitivity variance analysis, the ratio of water to gypsum has a very significant effect on *σ_c_*, *σ_t_*, *τ_f_*, *E*, *ν* and *c.* The content of iron powder has a highly significant effect on *σ_c_*, *σ_t_*, *τ_f_* and *c*, and it has a significant effect on *ν* and *φ.* The content of coarse sand has a significant effect on *σ_c_*, *E* and *c.*
(2) Except for *φ*, the multivariate linear regression model of other macro-mechanical parameters is highly significant. The fitting value of the regression model is similar to the curve shape of the orthogonal test results with a small error, which meets the test requirements of rock-like materials and can be used to predict the macro-mechanical parameters of rock-like materials in a certain range.
(3) In the process of inverse proportion optimization based on the genetic algorithm, the correct selection of materials, the correct application of experimental design methods and the appropriate selection of optimization models and algorithms are the keys to achieve good proportion optimization results. Through this program, it is concluded that the optimum proportion of rock-like materials with the closest mechanical properties to gneiss granite is a water–gypsum ratio of 0.5325, iron powder content of 3.975% and coarse sand content of 15.967%.

The research results of this paper aim to provide a genetic algorithm inversion matching optimization method for rock-like materials. Based on the macro-mechanical parameters obtained by the orthogonal test, the genetic algorithm is used to accurately and efficiently inverse the optimization results of the material ratio, which can provide certain reference value for the optimization design requirements of other similar materials. However, there are some limitations in this research process. In this paper, only the rock-like materials of gneiss granite were considered to optimize the proportion. The main analogy index is a 1/15 tension–compression ratio with typical brittle characteristics, which has not been extended to the value of a tension–compression ratio that characterizes more rock mechanical properties. In addition, in order to restore similar mechanical properties to the original rock characterization as much as possible, this paper considers selecting seven macro-mechanical parameters as investigation indicators to explore. This is our attempt to restore the mechanical properties of the target rock. Therefore, when using the multiple linear regression method to construct the quantitative formula of mechanical parameters and proportioning factors, nonlinear influencing factors are the main reason for the low determination coefficient. In particular, due to the difference in the properties of the two materials, *φ* shows strong nonlinear behaviour and cannot be analysed by multiple linear regression. At the same time, it will not be considered in the subsequent optimization research of rock-like materials based on genetic algorithm inversion. In the future, we plan to take the nonlinear influence of ratio factors on parameters as the main research object and further carry out related experimental research in order to seek better quantitative relationships and solve more practical problems in practical projects.

## Figures and Tables

**Table 1:** Rock-like material similarity scales and target macroscopic parameters.

Physical Quantity	Target Rock	Dimension	Similarity Relation	Similarity Ratio	Target Macro-Parameters
*σ_c_* (MPa)	80	*ML* ^−1^ *T* ^−2^	*S_σc_* = *S_ρ_S*_1_	1:5	16
*σ_t_* (MPa)	5.33	*ML* ^−1^ *T* ^−2^	*S_σt_* = *S_ρ_S*_1_	1:5	1.066
*τ_f_* (MPa)	19	*ML* ^−1^ *T* ^−2^	*S_τf_* = *S_ρ_S*_1_	1:5	3.8
*E* (GPa)	26	*ML* ^−1^ *T* ^−2^	*S_E_* = *S_ρ_S*_1_	1:5	5.2
*ν*	0.15	-	*S_ν_* = 1	1:1	0.15
*φ* (°)	58	-	*S_φ_* = 1	1:1	58
*c* (MPa)	11.5	*ML* ^−1^ *T* ^−2^	*S_c_* = *S_ρ_S*_1_	1:5	2.3
*σ_t_*/*σ_c_*	1/15	-	*S_σt_/_σc_* = 1	1:1	1/15

**Table 2:** Rock-like material ratio factors and research interval.

Rock-like Material Ratio Factors	Water-Gypsum Ratio	Iron Content (%)	Coarse Sand Content (%)
Research interval	0.46–0.54	2–4	14.3–23.8

**Table 3:** Orthogonal test factor level design.

Factor Levels	A	B (%)	C (%)
1	0.46	2	14.3
2	0.48	2.5	16.7
3	0.5	3	19
4	0.52	3.5	21.4
5	0.54	4	23.8

**Table 4:** Orthogonal test results.

Group	A	B (%)	C (%)	*σ_c_* (MPa)	*σ_t_* (MPa)	*τ_f_* (MPa)	*E* (GPa)	*ν*	*φ* (°)	*c* (MPa)
1	0.46	3	19	20.202	1.352	5.532	8.803	0.014	58.47	2.85
2	0.46	2.5	16.7	19.647	1.318	5.377	8.052	0.023	58.47	2.77
3	0.48	3	14.3	18.862	1.186	5.375	8.814	0.06	59.24	2.58
4	0.5	2	16.7	15.436	1.084	4.523	7.016	0.084	57.80	2.23
5	0.52	3	16.7	16.206	1.073	4.211	6.406	0.099	58.56	2.27
6	0.5	4	14.3	17.930	1.161	5.185	7.899	0.088	58.95	2.48
7	0.5	3	21.4	18.639	1.217	5.201	6.013	0.043	58.76	2.59
8	0.52	3.5	19	16.688	1.143	4.35	6.147	0.105	58.18	2.38
9	0.54	3.5	14.3	15.188	1.053	3.372	6.720	0.112	57.99	2.18
10	0.48	3.5	16.7	19.129	1.265	5.481	8.245	0.06	58.66	2.68
11	0.5	3.5	23.8	15.883	1.121	4.534	5.971	0.066	57.70	2.30
12	0.52	2	23.8	14.705	0.964	3.991	6.338	0.106	58.76	2.05
13	0.54	2.5	21.4	15.126	0.972	2.634	4.976	0.12	59.04	2.09
14	0.54	3	23.8	14.801	1.017	3.159	5.267	0.126	58.09	2.11
15	0.48	4	19	20.240	1.303	5.717	7.003	0.067	58.95	2.80
16	0.54	4	16.7	16.003	1.066	3.84	5.162	0.166	58.56	2.25
17	0.46	3.5	21.4	20.364	1.400	5.965	7.096	0.03	58.09	2.91
18	0.46	4	23.8	20.658	1.406	6.211	6.476	0.049	58.28	2.93
19	0.46	2	14.3	19.532	1.209	5.259	8.276	0.043	59.52	2.65
20	0.52	4	21.4	17.241	1.167	4.434	6.980	0.107	58.28	2.44
21	0.54	2	19	13.874	0.932	2.427	5.216	0.178	58.37	1.96
22	0.48	2.5	23.8	17.727	1.134	5.242	5.812	0.069	59.04	2.44
23	0.5	2.5	19	17.316	1.124	5.030	6.229	0.078	58.85	2.40
24	0.52	2.5	14.3	16.086	1.012	4.147	5.958	0.108	59.24	2.20
25	0.48	2	21.4	17.484	1.091	5.226	8.095	0.077	59.43	2.38

**Table 5:** The result of variance analysis.

Parameter	Significance Analysis	A	B(%)	C(%)
*σ_c_*	*F*	86.05	13.91	3.45
	*Sig.*	<0.001	0.009	0.042
	Degree	**	*	(*)
*σ_t_*	*F*	99.13	27.75	3.21
	*Sig.*	<0.001	0.005	0.052
	Degree	**	*	
*τ_f_*	*F*	71.12	5.84	0.09
	*Sig.*	<0.001	0.008	0.985
	Degree	**	*	
*E*	*F*	9.02	1.18	3.32
	*Sig.*	0.001	0.367	0.048
	Degree	**		(*)
*ν*	*F*	42.28	4.79	0.73
	*Sig.*	<0.001	0.015	0.588
	Degree	**	(*)	
*φ*	*F*	3.06	3.91	2.68
	*Sig.*	0.059	0.029	0.083
	Degree		(*)	
*c*	*F*	113.54	23.84	3.36
	*Sig.*	<0.001	0.006	0.046
	Degree	**	*	(*)

**Table 6:** Regression model collinearity diagnosis results.

Parameter		1	2	3	4
*σ_c_*	Characteristic root	3.935	0.274	0.197	0.044
	Conditional index	1.000	3.925	4.832	9.424
*σ_t_*	Characteristic root	2.962	0.208	0.036	-
	Conditional index	1.000	4.343	9.066	-
*τ_f_*	Characteristic root	2.971	0.362	0.033	-
	Condition index	1.000	2.968	9.687	-
*E*	Characteristic root	2.978	0.327	0.023	-
	Condition index	1.000	3.219	9.984	-
*ν*	Characteristic root	2.895	0.201	0.041	-
	Condition index	1.000	4.576	8.837	-
*φ*	Characteristic root	1.973	0.027	-	-
	Condition index	1.000	8.602	-	-
*c*	Characteristic root	3.896	0.289	0.163	0.045
	Condition index	1.000	3.821	5.903	9.384

**Table 7:** Multiple linear regression equation of macroscopic mechanical parameters.

Parameters	Multiple Linear Regression Equation	*R* ^2^
*σ_c_*	$y_{\sigma_{c}}=47.104-63.338x_{1}+0.937x_{2}-0.001x_{3}$	0.915
*σ_t_*	$y_{\sigma_{t}}=2.858-3.91x_{1}+0.083x_{2}$	0.919
*τ_f_*	$y_{\tau_{f}}=19.42-31.732x_{1}+0.368x_{2}$	0.903
*E*	$y_{E}=23.976-28.864x_{1}-0.146x_{3}$	0.708
*ν*	$y_{\nu }=-0.55+1.278x_{1}-0.002x_{2}$	0.821
*c*	$y_{c}=6.321-8.58x_{1}+0.152x_{2}-0.003x_{3}$	0.934

**Table 8:** Genetic algorithm inversion optimization calculation results.

Rock-like Material Ratio Factors	Water-Gypsum Ratio	Iron Content (%)	Coarse Sand Content (%)
Inverse result	0.5325	3.975	15.967

**Table 9:** Genetic algorithm inversion optimization results test.

Parameters	Required Value	Secondary Indoor Physical Test (*n* = 3)			Relative Error (%)
		Average Value	Standard Deviation	Coefficient of Variation	
*σ_c_* (MPa)	16	15.842	0.222	0.014	0.988
*σ_t_* (MPa)	1.066	1.055	<0.001	<0.001	1.043
*τ_f_* (MPa)	3.8	3.777	0.005	0.001	0.609
*E* (GPa)	5.2	5.128	0.585	0.114	1.385
*ν*	0.15	0.151	0.184	1.219	0.667
*c* (MPa)	2.3	2.375	-	-	3.158
*σ_t_*/*σ_c_*	1/15	1/15.016	-	-	0.107

## Data Availability

The data presented in this study are available on request the corresponding author.
